# A Novel Biosensor for the Detection of Glucose Concentration Using the Dual-Peak Long Period Grating in the Near- to Mid-Infrared

**DOI:** 10.3390/s24041247

**Published:** 2024-02-15

**Authors:** Namita Sahoo, Bing Sun, Yidong Tan, Kaiming Zhou, Lin Zhang

**Affiliations:** 1 Aston Institute of Photonic Technologies, Aston University, Birmingham B4 7ET, UK; k.zhou@aston.ac.uk (K.Z.); l.zhang@aston.ac.uk (L.Z.); 2 College of Electronic and Optical Engineering, Nanjing University of Posts and Telecommunications, Nanjing 210003, China; b.sun@njupt.edu.cn; 3 Department of Precision Instrument, Tsinghua University, Beijing 100084, China; tanyd@mail.tsinghua.edu.cn

**Keywords:** dual-peak long period gratings, enzyme immobilisation, glucose sensing

## Abstract

In this article, we demonstrate an improved efficient fibre sensor with a high sensitivity to measure glucose concentrations in the physiological range of human beings, operating in a broad spectral bandwidth from the near- to mid-infrared. The sensor consists of a dual-peak long period grating (DPLPG) with a period of 150 μm inscribed in an optical fibre with a diameter of 80 μm. The investigation of sensing for refractive index results in a sensitivity of ~−885.7 nm/refractive index unit (RIU) and ~2008.6 nm/RIU in the range of 1.30–1.44. The glucose measurement is achieved by the immobilisation of a layer of enzyme of glucose oxidase (GOD) onto the fibre surface for the selective enhancement of sensitivity for glucose. The sensor can measure glucose concentrations with a maximum sensitivity of −36.25 nm/(mg/mL) in the range of 0.1–3.0 mg/mL. To the best of our knowledge, this is the highest sensitivity ever achieved for a measurement of glucose with a long period grating-based sensor, indicating its potential for many applications including pharmaceutical, biomedical and food industries.

## 1. Introduction

Glucose plays a crucial role in maintaining human health by supplying essential nutrients for sustaining optimal brain performance and safeguarding muscles from malfunctioning. However, imbalance in blood glucose levels can cause a debilitating condition known as diabetes. In order to manage this condition effectively, continuous monitoring of blood glucose levels is essential. Several minimally invasive methods exist for detecting glucose level based on puncture tests, however these methods are associated with risks of infection. In contrast, an optical fibre sensor can be used in non-invasive ways and also exhibit reusability, providing a cost-effective and sustainable solution for long-term monitoring of blood glucose levels [1].

There are a variety of electrochemical sensors based on non-enzymatic type or wearable type [2,3], but these sensors have some drawbacks in terms of requirement of frequent calibrations, limited stability and selectivity. Optical sensors are advantageous over this for their high accuracy. 

The existing non-invasive optical methods which are associated with the light detection in the near infrared (NIR) range include different spectroscopy techniques. One of these is light scattering and absorption [4]. Here, the attenuation of reflected light depends on the human tissue depth and blood glucose concentration. Also, this affects the intensity for scattered light. The other technique is Raman spectroscopy [5,6], where Raman shift occurs when a NIR laser penetrates into the tissue and less fluorescence happens, allowing for the resolving of lower intense Raman spectrum. Along with this, glucose detection is possible in polarimetric methods by controlling the rotation of light polarisation through different concentrations of solutions [7]. The disadvantages with this method are associated with low specificity as the glucose containing fluids is contained with other active compounds such as ascorbate and albumin. On the other hand, photoacoustic spectroscopy [8] facilitates the amplitude detection of sound signals in the range of 1000–1800 nm by a NIR light absorption into the glucose sample. Another novel method known as optical coherence tomography (OCT), which is a high-resolution imaging method, uses low-coherence interferometry [9]. Here, biological tissue and blood vessels can be examined following the processes of functional imaging, monitoring and quantification. However, the limitations for all these detection techniques in the NIR range are of low coefficient regarding the absorption of glucose, have non-specific scattering coefficients and are influenced by chemical and physical parameters such as changes in blood pressure, body temperature, skin hydration and concentrations of triglyceride [10,11,12]. 

Optical fibre biosensors are one of the key areas in the field of biomedical applications leveraging novel optical structures and advanced coating materials and methods, which synergically contributes to their outstanding analysing capabilities for detecting and distinguishing various classes of biomolecules. Such a biosensor can be based on an optical fibre grating (OFG) which consists of a periodic perturbation of refractive index in the core of a single mode fibre. This structure diffracts light at phase matching conditions so that the light coupling occurs between the co-propagating fundamental core mode with the co- or counter-propagating core mode, cladding mode or radiation mode (leaky mode). Such gratings can be fabricated by irradiation of the photosensitive fibre with a high-power UV laser. Depending on its period, an OFG can be classified as a Bragg grating [13] or a long period grating (LPG) [14]. 

Noteworthily, the optical fibre sensing devices operating at mid infrared (mid-IR) range have shown interesting applications in defense [15], health [16,17] and environmental sensing [18]. Enhanced and highly sensitive detection capabilities are achievable in the mid-IR range spanning 2–20 μm. This spectral region is particularly advantageous for identifying characteristic fingerprint absorptions of numerous biomolecules, making it well-suited for precise and selective biomolecular detection [19,20]. Silica optical fibre can work up to the mid-IR spectral range. Sensors utilising these fibres and sensing in the mid IR near 2 μm can leverage an evanescent wave extended further outside the waveguide due to the lower refractive index of the material and longer wavelength. This extension enhances the sensors’ sensitivity, making them capable of achieving higher levels of detection precision. 

The conventional LPG generally has a period between 300–500 μm and is featured with a series of resonances over a wavelength span of 1200–1700 nm. These resonances correspond to coupling of light from the core mode to the cladding modes when the phase matched condition is satisfied. There have been reports on the dual-peak LPG (DPLPG) in the near infrared range. As such, Shu et al. demonstrated that LPGs with a period reduced to 160 μm begin to show a dispersion turning point in the spectrum, and the temperature sensitivity is increased significantly [21,22]. 

The dispersion turning point is characterised as the wavelength at which a pair of conjugate resonances converge. These resonances correspond to the coupling of the same-order cladding mode but exhibit opposite dispersion characteristics. The separated conjugated peaks can be observed in a wavelength range between 900–2300 nm. When the dual-peak resonances are in a proximity to the dispersion turning point, they become extremely sensitive to changes of many parameters, including temperature and surrounding refractive index. These types of LPGs have been termed as (DPLPG). 

The functionalisation of the surface of optical fibre with molecular reactive elements such as enzymes, deoxyribonucleic acids (DNA), antibodies and antigens has entailed the development of several bio-receptor immobilisation methods including covalent bonding [22], ionic bonding [23] and absorption [24]. The most effective method is covalent bonding, which stands out as the most effective method. It allows for the strongest, most uniform and chemically stable bonds.. Enzyme-functionalised sensors based on excessively tilted fibre grating (Ex-TFG) and normal LPG have been reported [25,26].

Here, we report the investigation of a suitable sensor device with a high sensitivity for glucose sensing. An enzyme, glucose oxidase (GOD), is used for selective conversion of glucose to gluconic acid, which can proportionally change the local refractive index subject to the level of glucose. As a result, it can detect very low glucose concentration in the range of 0.1–3.0 mg/mL. The high sensitivity is also ensured by a DPLPG inscribed in a fibre of thin cladding. 

Section 2 of the paper is dedicated to the theoretical analysis and characterisation of the DPLPG sensor. It begins with a device modelling in Section 2.1, followed by the revelation of the sensor’s sensitivity superiority in Section 2.2 for both temperature and surrounding refractive index. In Section 2.3, factors to consider for a device design that gives optimal performance are discussed. An experimental investigation on sensitivity is then presented. Section 3 of the paper is devoted to the sensing of glucose, which includes a detailed method for surface modification. Finally, the paper concludes with a summary of the findings and potential applicability.

## 2. Theory, Properties, Fabrication and Sensing Measurements of DPLPG 

### 2.1. Theory

**Figure 1:**
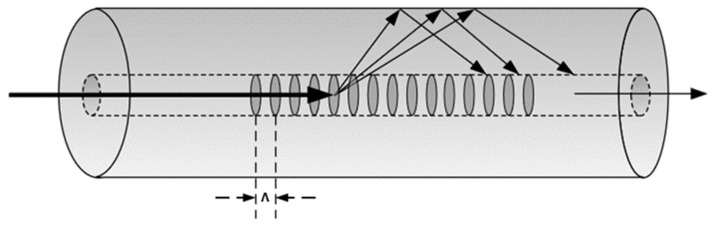
Schematic of the cladding mode coupling for a long period grating (LPG).

In a long period grating, the fundamental mode in the core is coupled with forward propagating cladding modes as depicted in Figure 1. A set of resonant bands can be found in the transmission spectrum and their central wavelength (*λ_m_*) is given by:

(1) $$\lambda_{m}=\left[n_{co}^{eff}-n_{cl,m}^{eff}\right]\Lambda =\partial n_{eff}\Lambda$$

Equation (1) discloses that *λ_m_* depends on the grating period (Λ) and $\partial n_{eff},$ which is the difference between the effective refractive index of the core mode ($n_{co}^{eff}$) and that of the *m*-th cladding mode $\left(n_{cl,m}^{eff}\right)$. A phase matching curve (Λ versus *λ_m_*) can be obtained based on Equation (1) and is generally monotonical. However, Shu et al. demonstrated in their work that when the period is small enough, the phase-matched curves can show a turning point where $\left|d\lambda_{m}/d\Lambda \right|\to \infty$. This is the dispersion turning point and the spectrum of the grating is extremely sensitive for surrounding changes [21]. The curves below the turning points have a positive dispersion of (d*λ_m_*/dΛ > 0) and have a negative dispersion of (d*λ_m_*/dΛ < 0). The spectrum of the LPG depends on the fibre structure and its surroundings. The effective refractive index of a cladding mode is a function of refractive indices of the fibre and the surrounding medium (*n_s_*). A change of *n_s_* can cause the wavelength to shift in the transmission spectrum.

The resonance wavelength of a LPG can be predicted theoretically by calculating the effective indices and electric field profiles of the core and cladding modes and determining the phase matching dispersion curves. Erdogan et al. developed an accurate theoretical formulation for this by considering a three-layer (core, cladding and ambient) cylindrical fibre structure and applying the coupled mode theory [27].

The transmission of the LPG for the attenuated band i is given by [21,28]

(2) $$T_{i}=1-\sin^{2}\left(\kappa_{i}L\right)$$

where *L* is the grating length and *κ_i_* is the coupling coefficient for the *i*-th cladding mode (*κ_i_*). These two parameters determine attenuation of the LPG at the resonant wavelength.

#### 2.1.1. Thermal Sensing Property

The thermal sensitivity can be found by differentiating the phase matching condition [29]. Therefore, the rate of change of the resonance wavelength with respect to temperature is given by

(3) $$\frac{d\lambda_{res}}{dT}=\lambda_{res}\cdot \gamma \cdot \left(\alpha +\Gamma_{temp}\right)$$

where *λ_res_* is one of the resonant wavelengths, *α* is the thermal expansion coefficient and *γ* is the dispersion factor defined as

(4) $$\gamma =\frac{\frac{d\lambda_{res}}{d\Lambda }}{n_{co}^{eff}-n_{cl,m}^{eff}}$$

The temperature dependence of the waveguide dispersion Γ*_temp_*, is given by:

(5) $$\Gamma_{temp}=\frac{\xi_{co}n_{co}^{eff}-\xi_{cl}n_{cl,m}^{eff}}{n_{co}^{eff}-n_{cl,m}^{eff}}$$

where $\xi_{co}$ and $\xi_{cl}$ are the thermo-optic coefficients of the fibre core and cladding materials, respectively [29]. The effective refractive index of the core is higher than that of the cladding, thereby the term ${(n}_{co}^{eff}-n_{cl,m}^{eff})$ is positive. 

Equation (3) tells that when the temperature of the grating increases, the resonant wavelengths of an LPG shift and their directions are dependent on fibre dispersion factor *γ*. For a normal LPG in standard SMF-28, the Λ − *λ* curve generally shows a positive dispersion in the spectrum range <1.7 μm when the cladding mode order is less than or equal to 7 (m ≤ 7). Thereby, the direction of peak shift is only dependent on the factor Γ*_temp_* as defined in Equation (5). Nevertheless, near the dispersion turning point, γ→∞ and thus the grating will show extremely large thermal sensitivity.

#### 2.1.2. Surrounding Refractive Index (SRI) Sensing Property

As part of the cladding mode extends to the vicinity of the fibre, the intensity and the wavelength of attenuation bands of an LPG can be modulated by the change of SRI (*n_sur_*). The SRI sensitivity, expressed as *dλ_res_*/*dn_sur_*, can be derived by analysing the effective index of cladding mode. It is given by

(6) $$\frac{d\lambda_{res}\;}{dn_{sur}}=\lambda_{res}\cdot \gamma \cdot \Gamma_{sur}$$

where Γ*_sur_* is the SRI dependent factor, defined as [29]

(7) $$\Gamma_{sur}=-\frac{u_{m}^{2}\lambda_{res}^{3}n_{sur}}{8\pi r_{cl}^{3}n_{cl}{(n}_{co}^{eff}-n_{cl,m}^{eff}){\left(n_{cl}^{2}-n_{sur}^{2}\right)}^{3/2}}$$

Here, *u_m_* is the *m*-th root of the zeroth order Bessel function of the first kind and *r_cl_* is the radius of fibre cladding. Therefore, the SRI sensitivity of LPG is determined by *γ* and Γ*_sur_*, which are dependent on the design parameters of the fibre and the grating and the mode number. From the detailed theoretical analysis by Shu et al. [21], the sensitivity of DPLPG and the direction of the wavelength shift depends on the coupling order of the cladding mode at the dispersion turning point.

As Equation (6) reveals, the sensitivity for the surrounding RI is proportional to γ (making it similar to that for the temperature) and becomes very large near the dispersion turning point.

### 2.2. Fabrication of the DPLPG

**Figure 2:**
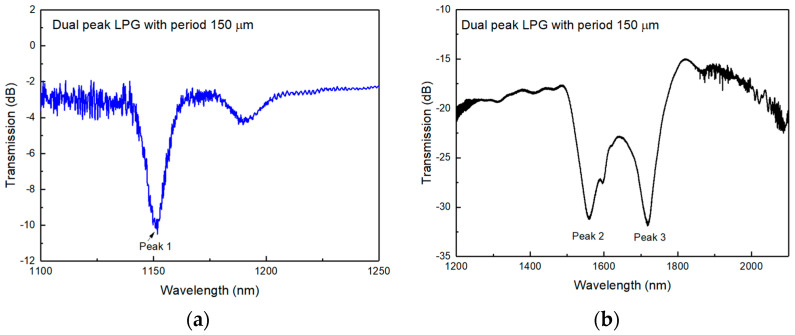
Transmission spectra for a DPLPG with 150 μm period UV-inscribed in SM1500 (4.2/80) fibre: (**a**) spectrum from 1100 nm to 1250 nm showing an individual peak, (**b**) spectrum from 1200 nm to 2100 nm showing the dual-peak feature of around 1650 nm area.

Since one of the paired peaks occurs in the wavelength range close to the mid-infrared 2 µm, the aim was to design and fabricate DPLPGs with resonance around 2 µm to be explored for environmental and biomedical sensing applications. Experimentally, DPLPGs were fabricated into a special small cladding B-Ge photosensitive fibre. The thin cladding fibre SM1500 (4.2/80) was bought from Nortel (Bell Northern Research, Kanata, ON, Canada). The DPLPGs with a period of 150 μm and the grating lengths between 10–15 mm were UV-inscribed. The typical transmission spectra of DPLPGs are shown in Figure 2a,b. In order to examine the spectral resonance from 950 nm to 2300 nm, a supercontinuum source of broad wavelength range covering the mentioned wavelength span and two optical spectrum analysers (OSAs HP and YOKOGAWA) was used to record the spectra for all the peaks.

The spectrum measurement reveals three major attenuation peaks including the dual-peaks near the dispersion turning point (1559.50 nm and 1718.80 nm, respectively). The other peak centred at 1151 nm corresponds to a resonance common to normal LPGs. Theoretically, each cladding mode would have two conjugate resonances if the spectral measurement ranges were sufficiently large. The DPLPGs are designed for its enhanced sensitivity near the dispersion turning point, and this unique property is evaluated for the temperature and refractive index sensing experiment.

### 2.3. Temperature Sensing and SRI Sensing and Analysing Results

**Figure 3:**
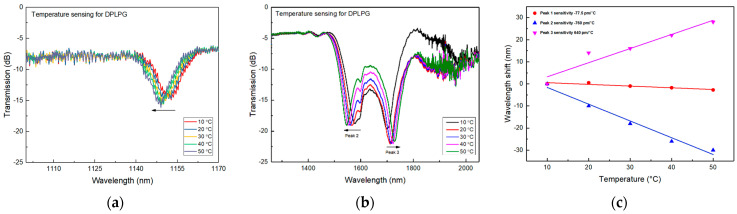
Temperature response in the range of 10–50 °C for a dualpeak LPG UV inscribed in the thin cladding SM1500 (4.2/80) fibre with a 150 μm period: (**a**) spectral evolution for the normal blue shifting peak 1, (**b**) spectral evolution for the dual-peaks 2 and 3 which shift in the opposite directions, (**c**) resonance wavelength versus increased temperature for all resonances.

The fabricated DPLPG was subjected to the temperature elevation experiment in the range of 10–50 °C with a 10 °C step. Same to the measurement mentioned in the earlier section, a supercontinuum source (950–2300 nm) was used to launch the light through the DPLPG. Two OSAs with spectral measurement range of 950–1200 nm and 1200–2300 nm were connected to the other end of the DPLPG to record spectral responses for peaks 1, 2 and 3, respectively. Figure 3a–c shows the thermal response for the three peaks. It can be clearly seen from the figures that the conjugated dual-peaks near the dispersion point (peak 2 and 3) move in the opposite directions with rising of the temperature. The sensitivity is much larger than peak 1, which is expected. The dual-peak resonances are showing the temperature sensitivities of −760 pm/°C for peak 2 at 1559.50 nm and 640 pm/°C for peak 3 at 1718.80 nm, respectively. The sensitivity for peak 2 appears negative due to the blue shifting of the attenuation resonance with an increase in temperature in the range of 10–50 °C. In contrast, following a similar trend, the normal single peak, which is far from the turning point, is showing much lower temperature sensitivity of only −77.5 pm/°C for peak1 at 1151 nm. It is clearly evident that the wavelength shift of the conjugated peaks near the dispersion point is very sensitive. Also noteworthy is the red shifting of peak 3 with increasing of temperature, which contrasts the other peaks. This is because factor in Equation (3) changes signs at the two sides of the dispersion turning point.

Further investigation of SRI sensing for the DPLPG was carried out by placing the device onto a glass plate and subjecting it to a series of SRI. Both ends of the fibre section containing the grating were fixed onto three-dimensional stages to neutralise the strain and bending effects. A series of commercial immersion gels with RI ranging from 1.30 to 1.44 (purchased from Cargille, Wayzata, MN, USA) were used for this experiment. The DPLPG was immersed in each immersion gel and the corresponding spectrum was recorded with an OSA resolution of 0.20 nm. It is seen that as SRI increases, peak 1 and 2 show blue wavelength shifts while peak 3 shows a red wavelength shift.

**Figure 4:**
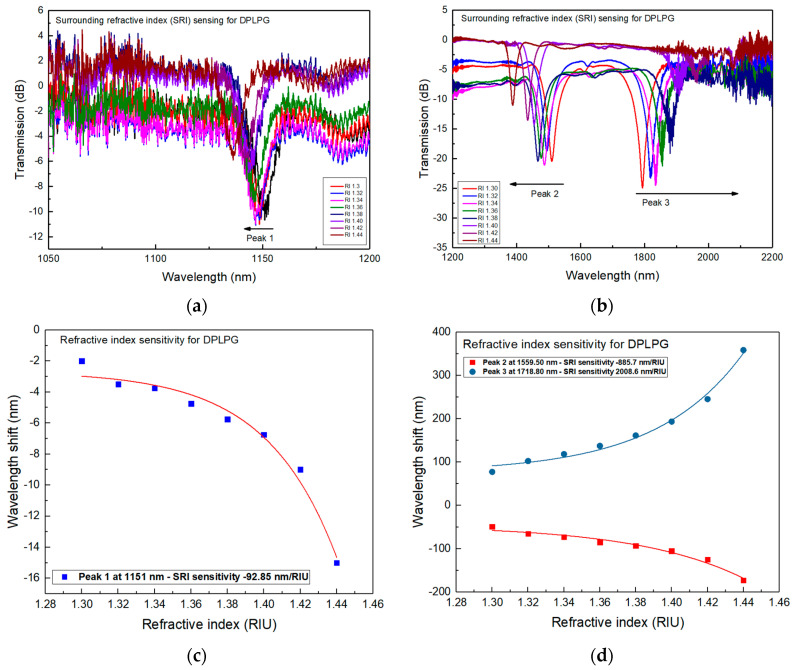
SRI response in the range of 1.30 to 1.44 for the DPLPG UV inscribed in the thin cladding SM1500 (4.2/80) fibre with a 150 μm period: (**a**) spectral evolution for the normal blue shifting peak 1, (**b**) spectral evolution for dual-peaks 2 and 3 which move in opposite directions, (**c**) SRI sensitivity result for peak 1 with increased SRI, (**d**) SRI sensitivity results for peak 2 and 3 with increased SRI.

The spectral evolution of the DPLPG when subjected to various SRI gels is shown in Figure 4a,b. From the figures we can see that the dual-peak resonances are showing opposite movement with increasing SRI. The first dual-peak (peak 2) blue shifts while the second (peak 3) red shifts. The individual peak at shortest wavelength is blue shifting with the SRI increasing but with a smaller rate. Figure 4c,d plot the SRI sensitivities for the three peaks. Considering the range of SRI from 1.30 to 1.44, the overall wavelength shifts are: −15 nm for peak 1, −171.2 nm for peak 2 and 355.72 nm for peak 3. The SRI sensitivity results show that the dual-peaks have much higher SRI sensitivities, reaching −885.7 nm/RIU for peak 2 and 2008.6 nm/RIU for peak 3. Similar to the temperature sensing, peak 2 of the DPLPG also exhibits a blue shift in resonance. This means that as the surrounding refractive index value increases, the wavelength becomes shorter. However, a very low sensitivity of −92.85 nm/RIU is obtained for peak 1 with its resonance shifting towards lower wavelength range. When the SRI changes from that of air (around 1.0) to 1.30, the sensitivities become much smaller, being −6.67 nm/RIU, −162.67 nm/RIU and 257.47 nm/RIU for the three peaks, respectively. Also, it is noted in the SRI range of 1.30–1.38, the DPLPG shows the maximum SRI responses: −550 nm/RIU for peak 2 and 1050 nm/RIU for peak 3, which is almost double that of peak 2. 

From the above results we can draw some important points: (i) the DPLPG have resonances close to the mid-infrared wavelength range, as the second resonance (peak 3) of the dual-peak is at 1718.80 nm; (ii) as they are so closed to the dispersion turning point, the dual-peaks are much more sensitive than the normal LPG single peaks, as the temperature and the SRI sensing results show their sensitivities are almost one order of magnitude higher. Therefore, DPLPGs could offer unique sensing functions for bio, medical and environmental sensing applications from the near- to mid-infrared range.

## 3. Surface Modification for DPLPG and Glucose Detection

### 3.1. Surface Modification for DPLPG Sensor

**Figure 5:**
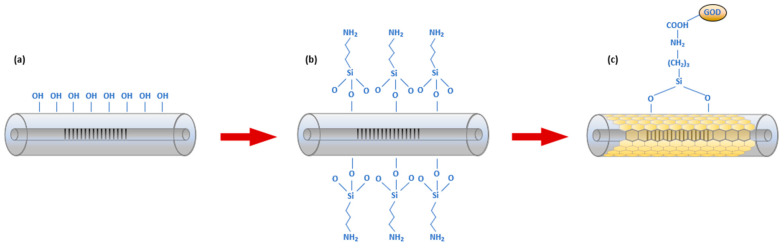
The functionalisation process for the DPLPG fibre sensor surface with (**a**) cleaning of the fibre, (**b**) APTES silanisation and (**c**) during and after GOD immobilisation.

Glucose oxidase (GOD) is an enzyme which enables the oxidisation of D-glucose to its corresponding lactone when sufficient oxygen is present. The GOD used for this experiment was procured from Sigma-Aldrich (St. Louis, MO, USA) with 50,000 units/g solid (without added oxygen). The other chemicals including D-glucose and a buffer solution of sodium acetate (SA) for pH 5.2 were purchased from Sigma-Aldrich. The grating surface modification with GOD involves several steps. Firstly, the fibre sensor was immersed into nitric acid (HNO_3_) solution (5% *v*/*v*) and heated to 40 °C by a hotplate for 2 h for the removal of any contamination on the sensor. Then it was washed with de-ionised water and ethanol multiple times. Afterwards, the cleaned fibre was immersed into sulfuric acid (H_2_SO_4_) solution (95% *v*/*v* in hydrogen peroxide (H_2_O_2_)) for 1 h at room temperature condition to activate the hydroxyl-groups (i.e., ‘-OH’) on the sensor surface. It was then dried at an incubated temperature of 40 °C for 18 h. Then the device was silanised with (3-Aminopropyl) triethoxysilane (APTES) (10% *v*/*v* into ethanol) solution. For this, the sensor device was immersed into the solution at room temperature for a duration of 40 min. In this process, the amine groups (NH^3+^ groups of the APTES molecules) would covalently link with the ‘-OH’ groups on the sensing surface. Thereafter, the fibre was cleaned with de-ionised water and ethanol several times to remove the non-covalently bonded silane compounds. A 10 mg/mL sodium acetate (SA) buffer solution was then prepared with GOD. Finally, the DPLPG device was immersed into the buffered solution of GOD for 2 h incubation, where the GOD’s ‘-COOH’ group would bind with NH^3+^ groups on the surface of the silanised fibre. Figure 5 shows the whole chemical reactions onto the fibre surface.

**Figure 6:**
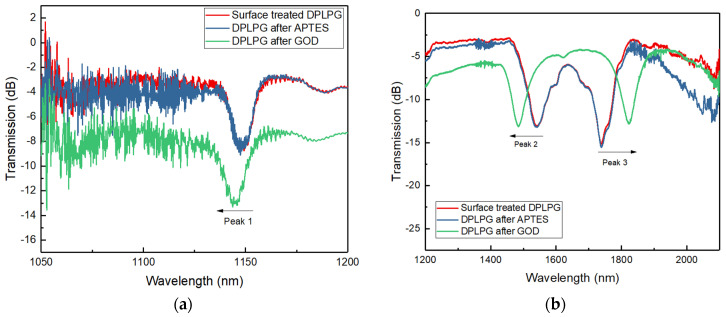
Spectral evolution for DPLPG after each process of surface treatment, silanisation with APTES and enzyme functionalisation with GOD for (**a**) lower wavelength peak 1 and (**b**) higher wavelength peaks 2 and 3.

The spectral property was examined in situ during the enzyme immobilisation process. Figure 6a,b show the resonance responses for all the attenuation peaks after each step of surface treatment process. It is seen from the figures that after the sensing device is surface treated and silanised with APTES, there is no observable wavelength shift of attenuation bands. However, after enzyme functionalisation with GOD, significant wavelength shifts are obtained for all the attenuation peaks. There are opposite wavelength shifts for two conjugated peaks 2 and 3 with the magnitudes of −45 nm and 86 nm, respectively. Peak 2 is blue shifted after the enzyme immobilisation. A comparatively small blue shift of ~−3 nm is observed for peak 1. These results clearly show that the dual-peaks are ultra-sensitive to SRI in comparison to peak 1.

### 3.2. Glucose Detection by the GOD-Immobilised DPLPG

The prepared GOD-immobilised DPLPG sensor device was used for glucose detection. In the experiment, the enzyme immobilised on the fibre surface of DPLPG selectively converts the glucose into gluconic acid, which would cause a refractive index to change in the vicinity of the fibre. The index change is proportional to the concentration of the glucose, and in turn it introduces a resonance wavelength shift of the grating sensor.

**Figure 7:**
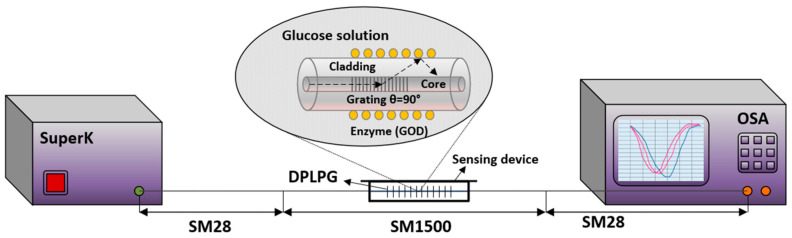
Schematic of the experimental setup for glucose sensing of enzyme-immobilised DPLPG.

Preparation of glucose solutions was performed with D-glucose (0.1–3 mg/mL) in the sodium acetate (SA) buffer. This provides a suitable chemical environment for this chemical reaction. The experimental setup for investigating the glucose sensing for enzyme immobilised DPLPG sensing device is shown in Figure 7. Light from supercontinuum source (Fianium, NKT Photonics, Birkerød, Denmark, 950–2300 nm) was launched into one end of the grating and the spectra were recorded using two optical spectrum analysers (OSA HP, HP Inc., Palo Alto, CA, USA, 950–1200 nm; and OSA YOKOGAWA, Yokogawa Electric Corporation, Tokyo, Japan, 1200–2300 nm) consecutively to cover all three resonance peaks from 1050 nm to 2300 nm (peak 1 in the lower wavelength range and peaks, peaks 2 and 3 in the higher wavelength range). The resolution of the OSA was set as 0.20 nm for both, and each sweep scan was considered between 30–60 s.

The SA-buffered solutions with various concentrations of D-glucose were prepared in vol/vol unit. In the process, firstly a maximum concentration of 3 mg/mL of D-glucose solution was prepared. Then, 2.5, 2.0, 1.5, 1.0, 0.5 and 0.1 mg/mL solutions were consecutively prepared by adding the required solvent to it. The measured refractive index value for the higher concentration was 1.3715 and reached 1.3704 for 0.1 mg/mL of glucose solution. Then the SRI sensing for the GOD immobilised DPLPG was carried out with the series of glucose solutions. The combined effect of a large number of D-glucose molecules and enough active GOD molecules existing in the evanescent field area led to the great change of $n_{cl}^{m}$, thus resulting in the wavelength shift of the DPLPG resonances.

**Figure 8:**
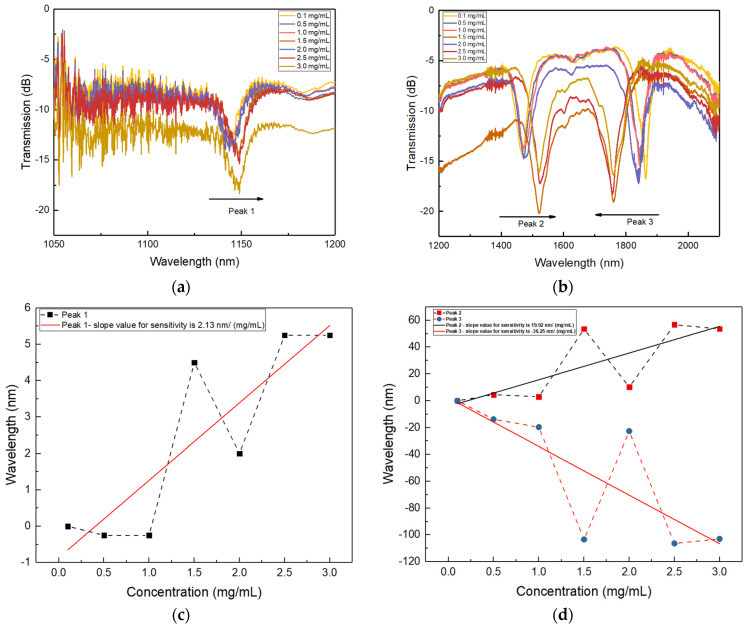
Spectral evaluation for enzyme functionalised DPLPG with various glucose concentrations for (**a**) resonance peak 1 and (**b**) resonance peaks 2 and 3. Sensitivity analysis for (**c**) normal single peak 1 and (**d**) conjugated attenuation peaks 2 and 3.

The plots in Figure 8a–d show the DPLPG resonance shift for different concentrations of glucose solutions and the sensitivity analysis results. It is seen in Figure 8a,b that the normal resonance (peak 1) shows a small red shift with an increase of the glucose concentration, whereas the conjugated attenuation peaks show opposite wavelength shifts. Further elaborated sensitivity plots with wavelength shift for different concentrations of glucose solution are depicted in Figure 8c,d. Here, the dashed line adjoining the data points reflects the true trend for sensitivity modulation of a DPLPG with respect to the different glucose concentration. It is clearly seen that there are opposite wavelength shifts at the dispersion turning points with gradual change in the refractive index of the surrounding medium. Non-linear behaviour may arise due to inaccuracies in measuring the concentration of solutions. The experimental setup used to prepare glucose solutions from higher-to-lower concentrations could potentially introduce inconsistencies, which leads to alterations of the refractive index qualitatively in some samples, rather than producing the precise values anticipated. Although, after considering all these facts, a linear fitting results in a sensitivity of 2.37 nm/(mg/mL) for peak 1 (as shown in Figure 8c) and the much larger sensitivities of 19.92 nm/(mg/mL) and −36.25 nm/(mg/mL) for peaks 2 and 3 at 1470.72 nm and 1863.28 nm, respectively (as shown in Figure 8d). As peak 3 shows the blue shift with the increased glucose concentration, its sensitivity is thus negative. The overall evaluated results clearly demonstrate that enzyme-functionalised dual-peak resonances are more sensitive to SRI in comparison with the normal peak 1. Henceforth, it paves the way for additional explorations into the applications of a highly sensitive DPLPG sensing device in the field of biomedical science.

## 4. Conclusions

In this paper, we have investigated and reported a precise glucose sensor inscribed into a thin-cladding optical fibre with a core-cladding diameter of (4.2/80) μm. A point-by-point writing technique was utilised to inscribe DPLPGs with the achieved resonances into the near-to mid-infrared (IR) range using a frequency-doubled Argon ion laser. We have demonstrated the thermal and surrounding refractive index (SRI) response for DPLPGs with very high sensitivity from near-IR to mid-IR range. The highest temperature sensitivity for peaks 2 and 3 are achieved as −760 pm/°C and 640 pm/°C, respectively. The high SRI sensitivities of ~−885.7 nm/RIU and ~2008.6 nm/RIU are achieved for the conjugate resonances of the DPLPG, respectively. Immobilisation of an enzyme, glucose oxidase (GOD), empowers this DPLPG device for precise sensing of glucose in the range of 0.1–3.0 mg/mL. In comparison with other reports on sensing of glucose, this work demonstrates a maximum sensitivity of −36.25 nm/(mg/mL) using the most sensitive dual-peak LPGs, which is applicable in biomedical sciences.

## Data Availability

The data are available on request.
